# *Notes from the Field:* Potential Infant HIV Exposure from Donated Breast Milk Received Through Social Media — Santa Clara County, California, May 2025–January 2026

**DOI:** 10.15585/mmwr.mm7531a2

**Published:** 2026-08-13

**Authors:** Alessandra Løchen, David Pang, Sonya Howard, Rene Padilla, Lizabell Monterrosa, Irene Sanchez Quintana, Kathy Klebba, Syeda Iqbal, Hailey Chua, Tommy Cline, Allison Aguilar, Brandon Bonin, Monika Roy, Akanksha Vaidya

**Affiliations:** ^1^County of Santa Clara Public Health Department, San Jose, California; ^2^Epidemic Intelligence Service, CDC.

**Summary**

**What is already known about this topic?**

HIV can be transmitted through breast milk; however, for mothers receiving antiretroviral therapy and who have a sustained undetectable viral load, this risk is <1%.

**What is added by this report?**

In October 2025, the County of Santa Clara Public Health Department investigated potential HIV exposure from donated breast milk received through a social media platform. An investigation identified five possibly exposed infants; the infants’ parents were encouraged to seek testing for them, as well as to obtain donated breast milk only from accredited milk banks. Investigators faced challenges in conducting the investigation through social media groups. No evidence of HIV transmission through the donated breast milk was identified.

**What are the implications for public health practice?**

Patient and public education about the risks associated with informal breast milk sharing through unregulated means and the availability of accredited milk banks might reduce potential exposures. Disease investigators could benefit from training in conducting investigations using official profiles in social media groups.

According to U.S. Department of Health and Human Services Perinatal HIV Clinical Guidelines, mothers with an HIV viral load of >50 copies/mL are recommended to not breastfeed because of HIV transmission risk. Exposed children are recommended to get tested for HIV immediately after the last exposure to breast milk, and at 14–21 days, 4–6 weeks, and 4–6 months after breastfeeding cessation (*1*,*2*). On October 17, 2025, the County of Santa Clara Public Health Department (SCCPHD) was notified of a woman who was 15 weeks and 4 days pregnant and who received a positive HIV test result at her prenatal screening on October 10. She reported that she was currently breastfeeding her daughter aged 2 years and donating breast milk through two social media groups.

## Investigation and Outcomes

An investigation was initiated by SCCPHD to 1) ensure that the mother and her daughter were linked to care, 2) identify and test contacts exposed to her breast milk, and 3) provide education to parents of exposed persons. This activity was reviewed by CDC, deemed not research, and conducted consistent with applicable federal law and CDC policy.*

The mother’s HIV viral load on October 27, 2025, before treatment, was 119,000 copies/mL. She had previously received a negative prenatal HIV test result on August 8, 2023, before her daughter’s birth. On October 30, 2025, she initiated antiretroviral therapy, disclosed that she was still breastfeeding her daughter, and was counseled to stop breastfeeding. Her daughter began receiving postexposure prophylaxis on October 31 and received a negative HIV RNA polymerase chain reaction (PCR) test result on November 3, 2025, and on January 13, 2026.

During May–October 2025, the mother had posted on social media groups offering to donate her breast milk. Investigators obtained information about social media groups to which she had offered breast milk and known families who acquired the breast milk. Investigators were prevented by group administrators from posting general messages in the groups; therefore, the investigators contacted all four families who had responded to the mother’s previous posts offering breast milk through direct messages in the social media platform using newly created individual profiles that named the health department or through contact information from electronic medical record searches and other public health database searches. Investigators used a combination of names, phone numbers, birthdays, or photos available on social media to match contacts to database records. The mother provided the name of one parent who received her breast milk, who was also contacted by investigators. Using responses to the mother’s posts on the social media groups, four other parents were identified as having infants who were at high risk for potential exposure; all five were contacted. Investigators asked parents if they had any remaining frozen donated breast milk, advised them not to feed their children any remaining frozen donated breast milk, encouraged them to seek testing for their children, and provided health education about using accredited milk banks to obtain safe, pasteurized breast milk.

Among the five identified contacted parents, one mother had kept the breast milk frozen and never used it. This milk was tested using HIV enzyme immunoassay and RNA PCR tests, which are not validated for breast milk; tests yielded positive results for HIV antibodies with a low level of virus (<30 copies/mL). Given the maternal seropositivity, detection of HIV antibodies in breast milk is expected and does not independently confirm infectious virus or transmission risk. Confirming whether the other four infants drank the donated breast milk was not possible; however, all four received negative HIV test results.

The investigators faced several challenges. These included 1) being accepted into the closed social media groups, 2) skepticism from contacted parents who questioned the legitimacy of the messages, and 3) being prevented from posting informational messages in the groups about safety risks related to sharing breast milk through social media groups.

## Preliminary Conclusions and Actions

Despite barriers to contacting parents who had received the donated breast milk, SCCPHD was able to identify and test infants at highest risk for potential exposure to HIV. Social media groups can be used to focus public health interventions toward persons informally exchanging breast milk that has not been properly screened. However, reaching these communities can be challenging, particularly because contact through social media can appear unofficial. Social media presents a novel means for disease investigation; health officials might benefit from additional training on how to conduct investigations through verified profiles in such groups.

Patient education regarding the health risks associated with unpasteurized breast milk obtained through social media groups or directly from another person can help mitigate potential exposures (*3*,*4*). Risks include contamination with viruses from women sharing their breast milk or bacteria from improper storage and handling or potential exposure to substances including prescription and nonprescription drugs that can be transmitted to the recipient (*4*). Screening and pasteurization are generally not available for breast milk received through social media groups that are not affiliated with accredited milk banks. Women unable to produce sufficient breast milk can safely obtain pasteurized donor human milk through these milk banks (*4*,*5*), which screen donors for infectious diseases and medication use and pasteurize the breast milk, providing a safe alternative for parents who want their children to be fed with breast milk.

## Footnotes

* 45 C.F.R. part 46.102(l)(2), 21 C.F.R. part 56; 42 U.S.C. Sect. 241(d); 5 U.S.C. Sect. 552a; 44 U.S.C. Sect. 3501 et seq.
